# High Resolution Mapping of Soil Properties Using Remote Sensing Variables in South-Western Burkina Faso: A Comparison of Machine Learning and Multiple Linear Regression Models

**DOI:** 10.1371/journal.pone.0170478

**Published:** 2017-01-23

**Authors:** Gerald Forkuor, Ozias K. L. Hounkpatin, Gerhard Welp, Michael Thiel

**Affiliations:** 1 West African Science Service Centre on Climate Change and Adapted Land Use—WASCAL, Burkina Faso; 2 University of Bonn, Institute of Crop Science and Resource Conservation (INRES), Soil Science and Soil Ecology, Nussallee 13, Bonn, Germany; 3 University of Wuerzburg, Remote Sensing Unit, Oswald-Kuelpe-Weg 86, Wuerzburg, Germany; Tennessee State University, UNITED STATES

## Abstract

Accurate and detailed spatial soil information is essential for environmental modelling, risk assessment and decision making. The use of Remote Sensing data as secondary sources of information in digital soil mapping has been found to be cost effective and less time consuming compared to traditional soil mapping approaches. But the potentials of Remote Sensing data in improving knowledge of local scale soil information in West Africa have not been fully explored. This study investigated the use of high spatial resolution satellite data (RapidEye and Landsat), terrain/climatic data and laboratory analysed soil samples to map the spatial distribution of six soil properties–sand, silt, clay, cation exchange capacity (CEC), soil organic carbon (SOC) and nitrogen–in a 580 km^2^ agricultural watershed in south-western Burkina Faso. Four statistical prediction models–multiple linear regression (MLR), random forest regression (RFR), support vector machine (SVM), stochastic gradient boosting (SGB)–were tested and compared. Internal validation was conducted by cross validation while the predictions were validated against an independent set of soil samples considering the modelling area and an extrapolation area. Model performance statistics revealed that the machine learning techniques performed marginally better than the MLR, with the RFR providing in most cases the highest accuracy. The inability of MLR to handle non-linear relationships between dependent and independent variables was found to be a limitation in accurately predicting soil properties at unsampled locations. Satellite data acquired during ploughing or early crop development stages (e.g. May, June) were found to be the most important spectral predictors while elevation, temperature and precipitation came up as prominent terrain/climatic variables in predicting soil properties. The results further showed that shortwave infrared and near infrared channels of Landsat8 as well as soil specific indices of redness, coloration and saturation were prominent predictors in digital soil mapping. Considering the increased availability of freely available Remote Sensing data (e.g. Landsat, SRTM, Sentinels), soil information at local and regional scales in data poor regions such as West Africa can be improved with relatively little financial and human resources.

## Introduction

Accurate and detailed spatial soil information is essential for sustainable land use and management as well as environmental modelling and risk assessment. In West Africa, where land degradation and loss in soil fertility has been reported by numerous studies [1–3], such information is increasingly required by governments and development partners to aid in improving land management [4]. High resolution spatial information on soils can assist decision makers to better target areas for soil fertility interventions and implement knowledge-based policies that aim at increasing agricultural production and improving livelihoods of small scale farmers in the sub-region. This is even crucial for the sustainable use of the soil resources particularly in the context of climate change [5].

Traditional soil mapping approaches have mostly relied on ground-based surveys. Classical field surveys including soil sampling and laboratory analyses are reported to be time consuming and expensive, especially when mapping is being done at national, regional or global scales [6–8]. In view of this bottleneck, new techniques for obtaining high-resolution soil information are being developed and still have to be optimized. Digital soil mapping, which incorporates secondary (non-soil) data sources into the mapping process, has been identified as a potential means of overcoming the limitations of traditional approaches and improving the detail and spatial coverage of soil databases [8–10]. Apart from its cost effectiveness, digital approaches allow a determination of an objective quantitative measure of prediction uncertainty, which is often not provided when traditional approaches are employed [11]. Remote Sensing (RS) data have come up in the last few decades as promising secondary data sources for improving digital soil mapping at all scales. Remotely sensed data sources: (1) contain extractable soil information, e.g. spectral reflectance, (2) have large spatial coverage and therefore permit mapping of inaccessible areas, (3) produce consistent and comprehensive data both in time and space and (4) offer possibilities of supplementing or at least reducing traditional soil sampling in soil surveys [12]. Based on these advantages, numerous studies have explored the use of RS data with varying spatial, temporal and spectral characteristics in digital soil mapping [8,13,14].

Saadat et al. [15] combined imagery from the Advanced Spaceborne Thermal Emission and Reflectance Radiometer (ASTER) sensor and a digital elevation model (DEM) for landform classification in Iran. They found that the spectral information in the RS data increased the possibility of distinguishing topographically similar landforms and subsequently improved the classification. Ehsani and Quiel [16] arrived at a similar conclusion when they used Landsat and Shuttle Radar Topographic Mission (SRTM) DEM for analysing landscape elements in Eastern Europe. Dobos et al. [6] found the combination of coarse resolution AVHRR (Advanced Very High Resolution Radiometer) data and DEM derived terrain derivatives to be promising data for characterizing soil forming environments and delineating soil patterns at national and continental scales. Compared to the terrain derivatives, the spectral information in the AVHRR bands were noted to have contributed more to the accurate delineation of soil types. Hahn and Gloaguen [17] underscored the importance of remotely sensed terrain variables (e.g. altitude, aspect, slope) as input to soil type classification in Germany. In a regional scale analysis, Scudiero et al. [18] found, among various variables, that surface reflectance of multi-year Landsat data was a useful indicator for characterizing the spatial variability of soil salinity in the western San Joaquin valley of California. Other studies also demonstrated the contribution of RS data in mapping soil properties such as sand, silt, clay and soil organic carbon (SOC) based on reasonable correlations between soil properties and reflectance spectra [14,19,20].

Despite many advances, further exploration of the application of RS data to soil mapping is required, especially in data poor regions such as West Africa. This is in light of the increasing availability of RS data, some of which are provided free of charge (e.g. Landsat, SRTM, Sentinel-1, -2) [8,21]. Research on the potential of RS data to improve digital soil mapping in West Africa is sparse [22]. Recent digital mapping initiatives on the continent (e.g. African Soil Information Service - http://africasoils.net/) [11] and at national scales (e.g. [23]) have used RS and other environmental variables in mapping soil units and properties. However, the spatial resolution of these studies is still coarse (ca. 250–1000 m), and may be of limited use for local scale (e.g. watershed) analysis. The derivation of digital soil data at local scales is important for assessing landscape scale resource needs and subsequently aid in regional, national and global soil and agricultural monitoring efforts [4,6]. Moreover, the success of digital soil mapping is to a large extent dependent on the availability, quality and timing of RS data acquisitions [24]. Land surface characteristics, especially on agricultural lands, are subject to temporal changes and it is not always clear which periods of the year are suitable for acquiring RS data for accurate soil property prediction. The use of multi-temporal images permits an investigation on the impact of the temporal window of RS data acquisition on prediction accuracies.

This paper reports findings of a digital soil mapping effort that integrated RS data and conventionally analysed soil samples to map the spatial distribution of soil properties (sand, silt, clay, cation exchange capacity, SOC and nitrogen) in a 580 km^2^ agricultural watershed in south-western Burkina Faso. High spatial resolution multi-temporal RapidEye and Landsat imagery together with ASTER Global DEM terrain derivatives were tested to determine their suitability for improving the availability and accuracy of spatial soil information in rural African landscapes. Since typical farm sizes in West Africa are small (i.e. less than one hectare [25]), the use of such high spatial resolution RS data for digital soil mapping at local scales is important and beneficial for optimizing farm management. However, such studies, to the best of our knowledge, are rare.

Four statistical methods which have proved their suitability for digital soil mapping in previous studies—multiple linear (MLR), random forest regression (RFR), support vector machine (SVM) and stochastic gradient boosting (SGB) [26–29] were explored to ascertain the most suitable method for high resolution RS data in the study region. Comparison of the traditional regression method (MLR) and different machine learning methods to spatially predict soil properties in West Africa are scarce. The research questions that the study addresses are: (1) which regression method offers the best accuracy for predicting soil properties? (2) What is the optimal time of RS data acquisition for predicting soil properties?

## Materials and Methods

### Study area

**Fig 1:**
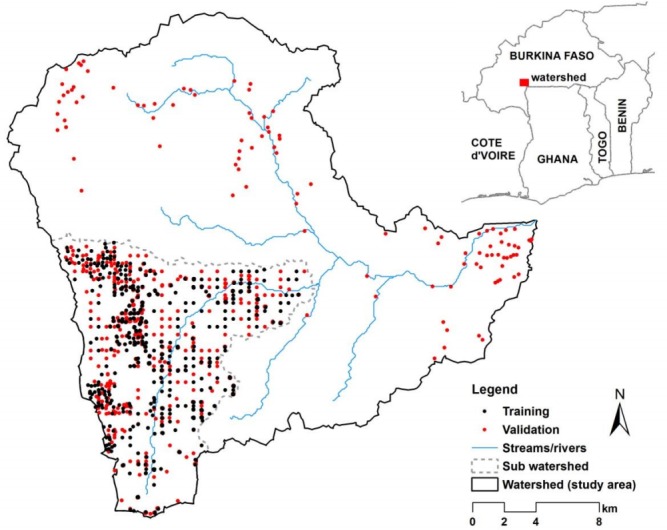
Map of the study watershed and locations of soil sampling.

The study was conducted in a rural watershed that falls in the Ioba province in south-western Burkina Faso (South-west Region). It has an area of about 580 km^2^ and lies between latitudes 11° 21’ 50” and 11° 04’ 27”N and longitudes 003° 08’ 37” and 002° 50’ 15”W (Fig 1). Detailed soil sampling was carried out in a sub-watershed which is about one-quarter of the watershed (Fig 1). The watershed has a uni-modal rainfall distribution (May-October), with an annual rainfall average of about 900 mm [30] while daily temperature ranges between 20.1 (minimum) and 34^0^ C (maximum). The lithology is composed of partly volcanic formations from the middle precambrian period and is made up mainly of andesic rocks with massive texture, basalt, diabase, gabbro and quartz-rich andesites. The study area is dominated by Plinthosols (70%) with inclusions of haplic Gleysols (15%), vertic Cambisols (14%), haplic Leptosols (1.7%) and stagnic Lixisols (0.2%). The terrain is flat, with elevation below 600 m above mean sea level and average slope of less than 5° [31].

Population density of the study area ranges between 40–60 persons/km^2^, most of whom engage in small scale rainfed farming as their main source of occupation [32]. Agriculture is the major land use and covers about 45% of the watershed, while different forms of vegetation (e.g. forest, shrub, grass), artificial surfaces (e.g. settlements, bare areas) and water bodies cover 52%, 2% and 1%, respectively [25].

### Input data

#### Soil sampling and analysis

Representative soil units were chosen for sampling based on existing soil [33], land use [34] and DEM [31] data of the watershed. The focus of the sampling was a sub-watershed (see Fig 1). A total of 1104 soil samples (1002 in sub-watershed and 102 outside) coming mainly from the topsoil (0–30 cm), were considered in this study. They were taken from the topsoil of 35 profiles along with intensive auger sampling carried out from July to October 2012 and from July to October 2013. At each auger sampling point, composite samples were taken from the topsoil (0–30 cm). These samples were dried at 40°C and sieved to 2 mm.

Because of the high number of soil samples, we analysed only 100 samples conventionally for the soil properties under study (i.e. texture–sand, silt, clay; nitrogen (N), SOC, cation exchange capacity (CEC)) following the procedures described in [35]. For the rest of the sample set, we used mid infrared spectroscopy (MIRS) to predict the above mentioned soil properties. The estimation of soil properties is generated by calibrating spectral information against conventionally obtained data using multivariate statistical procedures such as partial least squares regression (PLSR [36]).

For spectra measurement, about 20 mg of the soil samples were set into microplates and compacted with a plunger to get a level and plain surface in five replicates. The spectra were recorded using a Bruker Tensor 27 with an automated high throughput device (Bruker HTS-XT; Bruker Optik, Ettlingen, Germany). For each spectrum, 120 scans were recorded from 8000 to 580 cm^-1^ at a resolution of 4 cm^-1^ [37]. The software package OPUS QUANT (© 2006 Bruker Optik GmbH) was used for spectra analyses and for the prediction of soil properties with PLSR. OPUS QUANT uses a routine that automatically tests combinations of varying spectral ranges and data treatments for the optimum prediction power of the model. For each soil parameter, we conducted calibration procedures employing a leave–one–out, full–cross validation as well as a test-set calibration for checking model robustness as described by Bornemann et al. [37] (Table 1). The quality of the different models for each soil property was assessed based on their predictive ability with the R^2^, ratio of performance to deviation (RPD) and the standard error of prediction (SEP). For more technical information, readers are referred to [38]. Only models exhibiting good predictive ability (RPD>2) or close to that (RPD 1.7–2.0) [39] were used to make predictions for the remaining samples (Table 1). As seen in the table, the MIRS cross validation showed that SOC, followed by N presented the best prediction accuracy based on the R^2^ and the RPD. Additionally, the error metrics from the MIRS test-set validation confirmed the robustness of the different calibration models for all soil properties with R^2^ ≥ 80% and with RPD>2.

**Table 1:** Statistical parameters of the mid infrared spectroscopy-partial least squares regression prediction models (n = 100 samples) and of the predicted dataset (n = 1104 samples).

Parameters	Full cross-validation				Test-set validation (V = 10%)				Predicted dataset			
	R^2 (^%)	RMSECV	RPD	Slope	R^2^ (%)	RMSEP	RPD	Slope	Min	Max	Mean	SD
Sand (%)	70.5	6.8	1.8	0.7	80.9	5.7	2.5	0.7	3.5	66.2	37.6	9.6
Silt (%)	75.8	4.9	2	0.8	88.2	3.9	3	0.8	22.4	67.1	45.1	8.6
Clay (%)	77.6	6.2	2.1	0.8	80.6	5.5	2.4	0.8	0	54.5	20.8	8
CEC (cmolc kg^-1^)	75.6	3.6	2	0.8	90.5	3.2	3.6	0.8	0	36.3	12.5	5.9
SOC (%)	95.3	0.1	4.6	0.9	92.2	0.2	3.6	0.9	0	4.4	1.5	0.6
Nitrogen (%)	85.5	0	2.6	0.9	85.7	0	3	0.8	0	0.3	0.1	0.1

RMSECV: root mean square error of cross validation, RMSEP: root mean square error of prediction, RPD: ratio of performance to deviation, V: validation set, SD: standard deviation.

#### Satellite spectral data

Multi-temporal data from two optical sensors, RapidEye and Landsat, were used in this study. The images were acquired on 1^st^ March, 1^st^ April, 3^rd^ May 2013 (RapidEye) and 13^th^ June 2013 (Landsat). This period was selected to coincide with the peak of the dry season and the ploughing/planting period during which there’s little or no vegetation especially on croplands. RapidEye was obtained from the RapidEye Science Archive team of the German Aerospace Center (DLR) (https://resa.blackbridge.com/), while Landsat 8 was downloaded from the United States Geological Survey's GLOVIS website (http://glovis.usgs.gov). The RapidEye data has five spectral channels (blue, green, red, rededge and near infrared (NIR)) and a spatial resolution of 5 m (i.e. orthorectified, level 3A) [40], while Landsat has eleven spectral channels [41] and a spatial resolution of 30 m, which was later resampled to 5 m to ensure integration with the RapidEye data. Six out of the eleven spectral channels of Landsat (see Table 2) were used in the analysis. Images from both sensors were atmospherically corrected using the ENVI ATCOR software [42]. In addition to the original spectral bands, six soil and vegetation indices were calculated for each image. In all, twenty-one spectral bands and twenty-four spectral indices were derived (i.e. six indices for each of the four images). Table 2 provides further details of the spectral bands of RapidEye and Landsat as well as formulae and definitions of the spectral indices calculated. These spectral indices have been found to be useful in digital soil mapping [43].

**Table 2:** Spectral bands of satellite images used and definitions of soil and vegetation indices calculated.

**Spectral bands**							
	**Band number**	**1**	**2**	**3**	**4**	**5**	**6**
	**RapidEye band name**	Blue (B)	Green (G)	Red (R)	Red edge (RdE)	Near infra red (NIR)	
	**Landsat band name**	Blue (B)	Green (G)	Red (R)	Near infrared (NIR)	Shortwave infrared 1 (SWIR 1)	Shortwave infrared 2 (SWIR 2)
**Spectral indices**							
	**Indices**			**Formula**		**Index property**	**Reference**
	Brightness Index (BI)			((R^2^ + G^2^ + B^2^) / 3)^0.5^		Average reflectance magnitude	[43]
	Saturation Index (SI)			(R–B) / (R + B)		Spectral slope	[43]
	Hue Index (HI)			(2 * R–G–B) / (G–B)		Primary colors	[43]
	Coloration Index (CI)			(R–G) / (R + G)		Soil color	[43]
	Redness Index (RI)			R^2^ / (B * G^3^)		Hematite content	[43]
** **	Normalized Difference Vegetation Index (NDVI)			(NIR–R) / (NIR + R)		Health and amount of vegetation	[44]

#### Terrain and climatic variables

Terrain variables were extracted from the 30 m resolution ASTER GDEM (http://asterweb.jpl.nasa.gov/GDEM.ASP) [30]. Although previous studies have shown that the 90 m resolution SRTM DEM [45] has a superior absolute accuracy than ASTER GDEM [46], the latter was selected for this study due to its superior spatial resolution. Although the 30 m SRTM data has been made freely available, it came at a time that this manuscript was at an advanced development stage. The data was pre-processed to generate a depressionless DEM prior to the calculation of terrain variables. Climatic data (i.e. mean annual precipitation and temperature over 50 years) at 1 km resolution were obtained from worldclim [47].

In order to ensure integration with the RapidEye data, the DEM and climatic variables were resampled to 5 m resolution using the bilinear and bicubic interpolation methods, respectively. Table 3 lists the 29 terrain and climatic variables that were used in this study together with the relevant references. Most derivatives were calculated using the System for Automated Geoscientific Analysis (SAGA) software, while few were calculated with ArcGIS.

**Table 3:** Terrain and climatic variables considered in this study.

Parameters	Definition	Units		Authors
Slope*	Inclination of the land surface from the horizontal		Radians/%	[48]
Steepest slope	Maximal rate of elevation change ingravitational field		radians	[49]
Curvature	Curvature		degree m^-1^	[6]
General curvature	Combination of horizontal and vertical curvature		degree m^-1^	[26]
Plan curvature*	Horizontal (contour) curvature		degree m^-1^	[27]
Maximum curvature	Maximum Curvature		degree m^-1^	[50]
Minimum curvature	Minimum Curvature		degree m^-1^	[50]
Total curvature	Curvature of the surface itself		degree m^-1^	[51]
Parallel curvature	Parallel curvature		degree m^-1^	[52]
Rectangle curvature	Rectangle curvature		degree m^-1^	[52]
Flow line curvature	Flow line curvature		degree m^-1^	[52]
Profile Curvature	Vertical rate of change of slope		degree m^-1^	[53]
Horizontal curvature	Measure of flow convergence and divergence		degree m^-1^	[54]
Flow direction*	Path of water flow		-	[55]
Aspect	Direction the slope faces		degree	[53]
Cose Aspect	Direction the slope faces: eastness		Degree	[56]
Sine Aspect	Direction the slope faces: northness		degree	[56]
Elevation	Vertical distance above sea level		m	[53]
Protection index	Extent at which a cell is protected by relief based on the immediate surrounding cell			[52]
Topographic position index	Location higher or lower than the average of their surroundings			[27]
Saga Wetness Index	Ratio of local catchment area to slope		-	[57]
Flow accumulation*	Ultimate flow path of every cell on the landscape grid		-	[58]
Channel network base Level	Channel network base level elevation		m	[59]
Temperature (mean annual)	Temperature		°C	[60]
Precipitation (mean annual)	Precipitation		mm	[60]

The variables with (*) were calculated in SAGA as well as ArcGIS due to slight differences in the computational algorithms used by the two software packages.

### Models

#### Multiple linear regression (MLR)

Linear regression models aim at explaining the spatial distribution of a dependent variable by means of a linear combination of predictors (independent variables). In the case of this study, the various soil parameters are considered the dependent variables while the spectral and terrain/climatic variables are the independent variables. Linear regression models generally have the form:

$$y=a+\sum_{i=1}^{n}b_{i}\;*\;x_{i}\pm \varepsilon_{i}$$ (1)

where “*y*” is the dependent variable (soil parameter), “*x*_*i*_”are the predictors, “*n*” is the number of predictors, “*a*” is the intercept, “*b*_*i*_” are the partial regression coefficients and “*ε*” is the standard error of estimate. The regression equation is used to predict the spatial distribution of the parameter of interest based on the independent variables.

The “lm” function implemented in the R software [61] was used for MLR analysis. A matrix of predictors was developed by superimposing the training samples on the spectral and terrain/climatic spatial layers and extracting the corresponding values. One soil property was modelled at a time as the response (dependent) variable with the developed matrix as the predictors. For each model, the adjusted R^2^ and residual standard error were recorded. In addition, the predictors that were significant at 1% significance level were noted.

A common limitation of regression models is the problem of multicollinearity, which occurs when there is significant correlation between the predictors. Since the number of predictors identified in this study are many (seventy-four), and there could be high correlation between some of them, a stepwise regression analysis was first conducted to produce uncorrelated predictors needed to model each soil parameter and thereby minimize the problem of multicollinearity. Stepwise regression identifies a subset of predictors based on the statistical significance of the predictors (using stepwise, forward selection, or backward elimination) [62]. In this study, the “stepAIC” function as implemented in the “MASS” package [62] of the R statistical package was used for the stepwise regression. For each soil parameter, a subset of uncorrelated predictors were identified for subsequent analysis. Table 4 presents the number of spectral and terrain/climatic predictors that were eventually used in the MLR for each soil property. On average, less than 50% of the initial predictors were eventually selected for each soil property with the exception of carbon, for which 53% were selected. In order to ensure comparison with the Random Forest Regression (RFR), the same set of predictors were maintained for the RFR analysis, although it (RFR) does not greatly suffer from the multicollinearity problem.

**Table 4:** Number of spectral and terrain/climatic predictors used in modelling each soil parameter.

Data/Parameter	Sand	Silt	Clay	CEC	SOC	Nitrogen
Spectral	17	22	21	12	26	19
Terrain/climatic	9	10	5	13	12	12
Total	26	32	26	25	38	31

#### Random forest regression (RFR)

The RFR analysis was conducted using the “*Random Forest*” (RF) function as implemented in the RF package [63] of the R software [61]. RF [64] belongs to the family of ensemble machine learning algorithms that predicts a response (in this case the respective soil parameters) from a set of predictors (matrix of training data) by creating multiple Decision Trees (DTs) and aggregating their results. Each tree in the forest is independently constructed using a unique bootstrap sample of the training data. Whereas other machine learning algorithms (e.g. bagging and boostrapping [65]) use the best split among all predictors for node splitting, RF chooses the best split from a randomly selected subset of predictors. The introduction of this additional randomness decreases the correlation between trees in the forest, and consequently increases accuracy [66]. Additionally, RF requires no assumption of the probability distribution of the target predictors as with linear regression, and is robust against nonlinearity and overfitting, although overfitting may occur in instances where noisy data are being modelled [67]. For RF modelling, parameters requiring tuning such as the number of trees to grow in the forest (ntree) and the number of randomly selected predictor variables at each node (mtry) were set using the grid search method in the R “caret” package [68] using tenfold cross validation with 5 repetitions.

RF optionally provides information on the relative importance of the predictors (variable importance) used in the construction of the forest [63]. Two importance measures—%IncMSE and IncNodePurity—are frequently computed. To calculate %incMSE (increase in mean standard error), each tree is constructed with and without a predictor. Then, the difference between the two cases is averaged over all trees and normalized by the standard deviation of the differences. The second measure (IncNodePurity) represents the total decrease in node impurity from splitting on a predictor in the tree construction process, averaged over all trees. In RFR, the node impurity is measured by the residual sum of squares [63]. RF computes an internal accuracy measure based on the samples that are omitted from the bootstrapped samples used in the tree construction (i.e. out-of-bag, OOB). The accuracy of the model is given by the mean square error (MSE_OOB_) of the aggregated OOB predictions generated from the bootstrap subset and is computed as follows [63]:

$${\text{MSE}}_{OOB}=n^{-1}\sum_{i=1}^{n}{\left(z_{i}-{\hat{z}}_{i}^{OOB}\right)}^{2}$$ (2)

Where “n” is the number of observations, z_*i*_ is the average prediction of the ith observation and ${\hat{z}}_{i}^{OOB}$ is the average prediction for the ith observation from all trees for which the observation was OOB.

The explained variance is expressed as follows:

$$\text{Var}=1-\frac{MSE_{OOB}}{Var_{resp}}$$ (3)

For each soil parameter modelled, the MSE_OOB_ explained variance and variable importance measure were recorded for subsequent analysis and discussion.

#### Support vector machines for regression (SVM)

Initially used for classification, the support vector machine (SVM) has been extended for regression with the prediction of soil properties [28,69]. Relying on Kernel functions, input data are plotted into a new hyperspace where separations are performed. The ultimate purpose is to get an optimal hyperspace for data fitting and prediction using the ε-insensitive loss function, which tolerates errors smaller than the constant ε set as a threshold. Detailed information about SVM can be found in Hastie et al. [70]. The determination of the best parameters (bandwidth cost parameter, insensitive loss function,) for tuning the model for each soil parameters was carried out using the grid search method in the R “caret” package [68]. For this purpose, ten random partitions of the training data with five repetitions was carried for leave-one-group-out cross-validation of the model. Parameters resulting in the lowest root mean square error were considered for modelling.

#### Stochastic gradient boosting (SGB)

Stochastic gradient boosting (SGB; [71,72]) is a hybrid method incorporating both boosting and bagging approaches. First, small classification or regression trees are sequentially built from the residuals of the preceding tree (s). Instead of focusing on the full training set, the SGB carries out a boosting by selecting (without replacement) at each step a random sample of the data leading to a gradual improvement of the model. More details related to the background and mathematical functions behind the SGB can be found in Ridgeway [73]. The required parameters for model fitting (interaction depth, shrinkage rate) were set by using the tenfold cross validation with five repetitions also with the R “caret” package [68]. For each soil property, parameters with the lowest error metric (root mean square error) were used for the final model.

### Accuracy assessment

The performance of the four models–MLR, RFR, SVM, SGB–in predicting the soil properties was assessed by using 80% of the detailed soil samples in the sub-watershed (which was the focus of the sampling) (Fig 1) for cross validation. A 10-fold cross-validation scheme with 5 repetitions was applied to ensure model stability and reliability using the “caret” R Package [68]. The remaining 20% served as an independent validation dataset. In order to assess the predictive strength of the models outside the sub-watershed (i.e. the core sampled area), all the soil samples outside the sub-watershed (102 samples) (Fig 1) were reserved for the purposes of accuracy assessment and used as a second independent validation dataset.

Though R^2^ is a valid statistic for assessing the prediction accuracy of a model, a high R-squared model may not necessarily lead to accurate predictions. This is because the model could systematically and significantly over- and/or under-predict the data at different points along the regression line. An over-fitted model could also lead to poor predictions [74]. It is, therefore, important to evaluate the models with other performance statistics, preferably based on an independent set of observations, to provide additional information on the prediction accuracy of the models.

For each soil parameter, two error statistics—root mean squared error (RMSE) and the symmetric mean absolute percentage error (sMAPE)—were calculated (see Eqs 4 and 5). The two statistics served as the basis for comparing the performance of the two models in predicting the spatial distribution of the different soil properties. Although RMSE is a frequently used statistic in the literature to indicate the average error of a model [75], its dependence on scale makes it difficult to calculate a model’s error in percentage terms. The sMAPE [76], on the other hand, provides a percentage-wise error and facilitates a comparison of the accuracy with which each soil property is predicted. The sMAPE (as defined in this paper), however, can provide unreliable estimates if either observed or forecasted value is negative [70].

$$RMSE={\left[\frac{1}{n}\sum_{i=1}^{n}{\left(P_{i}-O_{i}\right)}^{2}\right]}^{1/2}$$ (4)

$$sMAPE=\frac{1}{n}\sum_{i=1}^{n}\frac{\left|O_{i}-P_{i}\right|}{(O_{i}+P_{i})/2}$$ (5)

where “P” is the predicted value and “O” is the observed/true value.

## Results and Discussion

### Model performance

The performance of the four models investigated was assessed based on: (1) model internally generated accuracy statistics and (2) independent validation samples.

#### Assessment based on internal accuracy statistics

This assessment was achieved by comparing the RMSE and the adjusted R^2^ (hereinafter referred to as R^2^) derived from the four models for the respective soil parameters. Table 5 presents results of the comparison. R^2^ ranged between 21 and 53% for MLR, 18 and 53% for RFR, 20 and 51% for SVM and 16 and 51% for SGB. Silt was the only soil parameter that achieved an R^2^ of greater than 50% for all models. The other soil parameters recorded relatively lower R^2^, with sand, clay, SOC and nitrogen consistently having R^2^ below 40%. The generally low R^2^ obtained in this study independently of the models can be attributed to a complex interplay and high variability of environmental factors in the studied watershed and surrounding regions [12,77]. High variability in agricultural soil management practices, nutrient application, vegetation cover and climatic factors (temperature, precipitation) are believed to be among the factors that resulted in the low correlations observed. Nonetheless, the range of R^2^ values obtained in this study is comparable to other studies that considered only terrain/climatic covariates [26,77] or only spectral data [43,78].

**Table 5:** Internal model validation based on 80% training data (All Spectral and topographic/climate predictors).

Model	Sand		Silt		Clay		CEC		SOC		Nitrogen	
	RMSE	R^2^	RMSE	R^2^	RMSE	R^2^	RMSE	R^2^	RMSE	R^2^	RMSE	R^2^
MLR	7.566	0.346	5.940	0.537	6.946	0.212	4.786	0.357	0.546	0.348	0.038	0.352
RFR	7.586	0.342	5.937	0.538	7.022	0.185	4.689	0.383	0.528	0.39	0.038	0.354
SVM	7.592	0.342	6.091	0.519	6.993	0.206	4.889	0.333	0.551	0.341	0.038	0.339
SGB	7.707	0.318	6.094	0.514	7.164	0.162	4.767	0.360	0.539	0.367	0.038	0.339

Table 5 shows that RFR performed marginally better than the other models in generating a model for the soil parameters with relatively lower RMSE and higher R^2^. The only exception was in the case of sand and clay, where MLR performed better than the RFR recording better error metrics. Generally, the machine learning methods (RF, SVM, SGB) were found to be more accurate than MLR using the RSME of cross validation for assessing model performance [79,80].

#### Assessment based on independent validation samples

Tables 6 and 7 present model performance statistics for the external validation inside (20% of the dataset) and outside the small catchment, respectively (see Fig 1). Here, the symmetric mean absolute percentage error (sMAPE) (Eq 5) was calculated and used as the basis for comparing the four models. Inside the small catchment, the RFR generally performed better than the other models, achieving the highest prediction accuracy (i.e. 100-sMAPE) for four soil properties (sand, silt, SOC, nitrogen) while SVM and SGB produced the best prediction for clay and CEC, respectively. Prediction accuracies by the RFR model ranged from a low of 68% for CEC to a high of 90% for silt, with an average accuracy of 77%. Compared to the MLR, for example, RFR improved prediction accuracy by 0.9% for sand, 0.4% for silt, 9.7% for CEC, 2.4% for SOC, and 1.7% for N. Generally, SVM and SGB also outperformed the MLR. In assessing the models’ performance outside the small catchment, Table 7 reveals that RFR achieved a better prediction accuracy for silt (85%) and clay (52%), SVM for sand (81%) and SOC (53%), and SGB for CEC (60%) and nitrogen (55%) with prediction accuracies of 69%, 85%, and 52%, respectively. The RFR model achieved an average accuracy of 62% for the validation outside the small catchment.

**Table 6:** External validation in small catchment based on 20% testing data with spectral data and terrain/climatic variables.

Model	Sand		Silt		Clay		CEC		SOC		Nitrogen	
	RMSE	sMAPE	RMSE	sMAPE	RMSE	sMAPE	RMSE	sMAPE	RMSE	sMAPE	RMSE	sMAPE
MLR	8.482	0.189	5.900	0.107	6.708	0.239	4.787	0.415	0.541	0.285	0.043	0.290
RFR	7.764	0.180	5.708	0.103	6.590	0.242	4.593	0.318	0.512	0.261	0.041	0.273
SVM	8.415	0.188	5.899	0.107	6.667	0.234	4.897	0.394	0.549	0.283	0.043	0.287
SGB	7.954	0.189	5.819	0.107	6.791	0.242	4.562	0.314	0.526	0.272	0.041	0.286

**Table 7:** External validation based on 102 samples outside the small catchment with spectral data and terrain/climatic variables.

Model	Sand		Silt		Clay		CEC		SOC		Nitrogen	
	RMSE	sMAPE	RMSE	sMAPE	RMSE	sMAPE	RMSE	sMAPE	RMSE	sMAPE	RMSE	sMAPE
MLR	17.341	0.547	9.350	0.157	11.804	0.548	5.597	0.469	0.847	0.505	0.059	0.496
RFR	14.115	0.314	8.713	0.146	10.623	0.478	4.891	0.415	0.765	0.472	0.053	0.457
SVM	20.257	0.193	9.106	0.153	14.738	0.566	5.669	0.448	0.750	0.471	0.057	0.488
SGB	15.184	0.341	8.846	0.148	10.875	0.497	4.960	0.398	0.759	0.476	0.051	0.454

Compared to MLR, the high performance of RFR and the other machine learning models could be due to the existence of a non-linear relationship between soil parameters and the predictors which MLR could not adequately resolve. Although MLR is widely used in statistical predictions, its limitation in handling non-linear relationships between response and predictor variables, especially in heterogeneous landscapes, has been noted in literature [74,81,82]. Non-parametric models such as RFR, SVM and SGB have been found superior to MLR due to their ability to handle non-linear relations and multi-source data [17,80,83]. In general, many studies reported RFR as providing better predictions compared to SVM [29,84–86]. However, Were et al. [87] found SVM as best predictor for the spatial distribution of SOC stock compared to RFR. Rossel et al. [88] reported RFR as having better prediction accuracy compared to SGB, while Hitziger et al. [89] found the latter superior to the former in soil property prediction. Similarly, SVM and SGB occasionally outperformed RFR in this study. This, and previous results, suggest that no single machine learning algorithm might serve best for every landscape and that many models should be calibrated to identify the most accurate model for prediction.

A comparison of Tables 6 and 7 reveals a general reduction in the predictive accuracy of the models outside the small catchment (which was the focus of sampling), although the magnitude of reduction varies depending on the model and soil property. Taking RFR, for example, the magnitude of reduction in prediction accuracy (i.e. 100-sMAPE) equalled 13% for sand, 4% for silt, 24% for clay, 10% for CEC, 21% for SOC, and 18% for nitrogen. In general, all models performed relatively poor in predicting clay, SOC and nitrogen outside the small catchment, with average accuracy reductions of 28%, 20% and 19%, respectively. On the other hand, the models performed well in predicting silt and CEC outside the small catchment, showing minimal accuracy reductions of 4% and 7%, respectively. These results suggest that the accuracy of extrapolating soil predictions outside the sampled area may differ depending on the soil property as well as on the non-comparability of the small catchment with regard to surface, land use and other characteristics.

Despite these differences, the accuracies achieved in the external validation can be assumed to be reasonably good considering the heterogeneity and size of the watershed in this study. Barnes and Baker [14] noted that the use of multi-spectral data for predicting the spatial distribution of soil properties can achieve optimal results when the study is conducted in an area with uniform soil surface characteristics. Consequently, several of such studies have been conducted at plot level or on relatively small watersheds [19,43,81], apparently to reduce the effect of varying surface characteristics.

Based on their study within a 350 ha demonstration farm in Arizona, Barnes and Baker [14] found that variations in surface characteristics such as crop residue, soil moisture and row orientation between fields limited the accuracy with which soil properties were mapped. These differences in surface characteristics may have influenced the results of this analysis, considering that the study area is an agricultural watershed populated by smallholder farmers who use diverse farm management practices [32,34]. The mode and time of land preparation (e.g. tractor, bullocks, manual) [90], nutrient application (e.g. fertility) [91] and water management strategy [92] can differ to a high degree from field to field due to availability of labour, crops to be cultivated or farm inputs utilized. Model calibrations based on samples from such localized and highly variable conditions can limit its predictive capacity outside the sampled areas [19,93].

Limited accuracy could also be related to potential error propagation from the MIRS models to the maps. Digital soil mapping based on mid infrared spectroscopy—partial least squares regression (MIRS-PLSR) prediction models might be affected by uncertainties at varying level of the mapping process such as spectra collection, model building and resulting prediction. Due to the heterogeneity of the landscape both in the small catchment and even more in the bigger catchment all the spectral variability might not have been covered resulting in possible feedback on the accuracy of MIRS-PLSR prediction models. Based on the classification of MIRS models by Reeves and Smith [94], the MIRS-PLSR calibration models in the present study (Table 1) range from models with very high predictive ability as for SOC (R^2^ = 95%, RPD = 4.6) to models with high (R^2^ = 85%, RPD = 2.6) to medium predictive ability (R^2^ = 70–77%, RPD = 1.8–2.1) respectively for Nitrogen and for the remaining soil properties (CEC, sand, silt and clay). In some other studies, MIRS provided better prediction models for SOC, N, CEC (R^2^ > 0.77) compared to clay, silt and sand (R^2^ = 0.22–73%) [95,96]. Though uncertainty propagation analysis as carried out by Brodský et al. [97] was out of the scope of the present study, the error metrics from the test set validation provided satisfactory evidence on the predictive ability of the MIRS-PLSR models (R2 > 80%, RPD ≥ 2). These results indicated that the calibrations were consistent especially for SOC, CEC, N and silt (R2 > 85%, RPD ≥ 3). In their study, Brodský et al. [97] found PLSR (with visible and near infrared) to cause lower uncertainties in the final map compared to uncertainty originating from ordinary kriging used as mapping model. Based on the sMAPE, the RFR and remaining machine learning models displayed quite satisfactory accuracy from the prediction of MIRS-PLSR models. This is obviously to their ability to handle both linear and non-linear patterns in dataset.

### Variable importance and temporal window for acquisition of RS data

The 5 top spectral and terrain/climatic variables which contributed most to the accuracy of digital soil mapping in the studied watershed are discernible from Table 8. Though RFR generally provided better predictions, variable ranking from the MLR model was included in the table for comparison purposes. The data in Table 8 reveal that both models include elevation in the list of the five most significant predictors for SOC and N while the other soil parameters had only spectral predictors. The only exception was for clay for which the RFR recorded also temperature among its driving factors while the MLR also displayed precipitation as key factor following elevation.

**Table 8:** First five predictors that were highly significant for RFR (based on “IncNodePurity” importance measure) and MLR analysis.

Model	Rank	Sand	Silt	Clay	CEC	SOC	Nitrogen
MLR	1	june_SWIR2	june_SWIR2	june_NIR	june_SWIR2	Elevation	Elevation
	2	june_green	June_RI	June_RI	May_RI	prep	March_NDVI
	3	June_CI	may_red	may_blue	may_RE	march_NIR	march_NIR
	4	may_green	june_red	June_SI	June_BI	March_NDVI	march_green
	5	April_HI	June_BI	June_CI	june_red	june_SWIR1	March_CI
RFR	1	june_SWIR2	June_RI	june_NIR	june_SWIR2	june_red	june_NIR
	2	may_NIR	May_SI	June_RI	june_blue	june_NIR	June_SI
	3	june_green	june_SWIR1	june_blue	May_RI	Elevation	Elevation
	4	May_SI	june_SWIR2	june_SWIR1	March_NDVI	June_SI	march_green
	5	may_green	May_CI	temp	june_red	June_BI	may_red

The names of the spectral predictors (see Table 2) here are a concatenation of the month of satellite acquisition and a spectral channel or indice. For example, “May_BI” represents the brightness index calculated from the May RapidEye image. prep: precipitation, temp: temperature.

Similar to the findings of this study, Hengl et al. [11] also recorded elevation as the most important variable influencing SOC contents of topsoil in Africa. Wang et al. [98] found that elevation and slope, along with soil clay and water contents, were among the most significant factors affecting SOC and N variability. Terrain/climatic variables are reported to have control on soil water status, dynamics of plant litter mineralisation as well as erosion and deposition processes [11,98]. The influence of elevation on predicting SOC and N, for example, can be related to corresponding variations in soil temperature as well as the intensity of cultivation which is higher in the lower areas as compared to the higher areas because of accessibility.

Table 8 reveals that generally, satellite images acquired in June and May were the most important in developing a model for predicting the soil properties under consideration. Spectral bands of the June Landsat image consistently came up as important predictors for the soil properties. The prominence of June and May images can partly be explained by the coincidence with the ploughing period or early stages of crop development when the soils of most agricultural plots are exposed. This allows satellite sensors to directly measure soil reflectance; hence, a good correlation between laboratory processed soil samples and satellite derived spectral reflectance is possible. The March imagery was the most important spectral predictor for SOC and N in MLR and was listed also for CEC and N in RFR (Table 8). March and April are the hottest months in the studied watershed, thus the prominence of the March imagery could be attributed to a higher loss of biomass with consequent higher mineralisation rate and SOC input.

Table 8 further reveals that the shortwave infrared (SWIR) and near-infrared (NIR) channels of Landsat, as well as soil specific indices like brightness, redness and saturation index were important spectral predictors in developing the respective models. The importance of the SWIR and NIR channels in this analysis confirms the findings of other studies. Liao et al. [99] used Landsat ETM bands as covariates in modelling soil textural properties (sand, silt, clay) and found that NIR (band 4) and SWIR (band 5, band 7) had a significant correlation with the analysed soil properties and explained most of their variability. Soil specific spectral indices were also found useful in digital soil mapping by other studies [43].

### Maps of the spatial distribution of the soil properties

**Fig 2:**
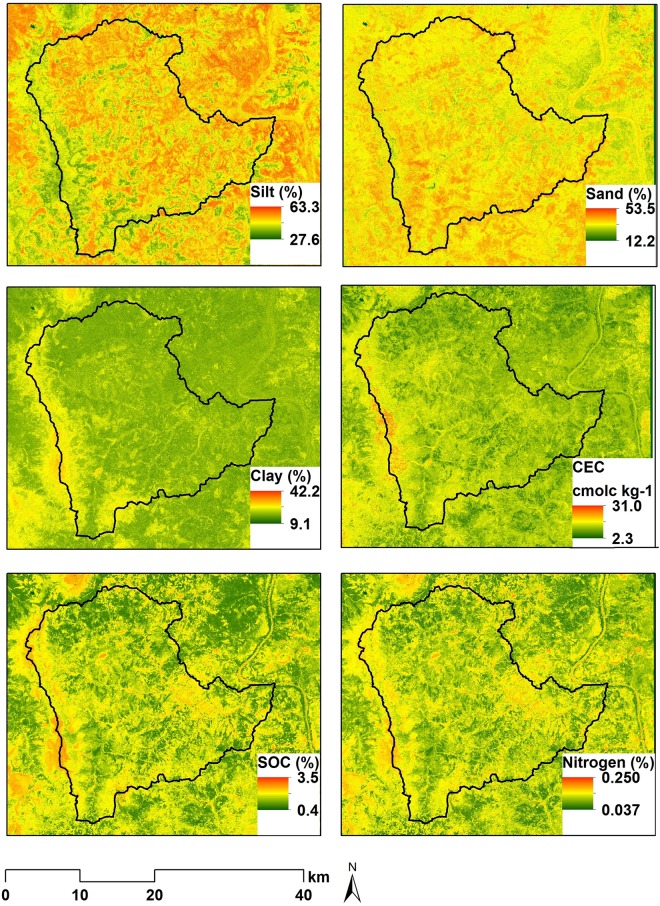
Spatial distribution of sand, silt, clay, cation exchange capacity (CEC), soil organic carbon (SOC) and total nitrogen (N) in the topsoil of the studied watershed.

In our study, the spatial distribution of soil properties does not display a clear pattern of hot and cold spot areas for all soil properties, but rather a patchy distribution (Fig 2). However, along the western border of the study area, medium to higher values of clay, CEC, SOC and N are observed as evidenced by a continuous yellowish band and reddish spots on the boundary line while the proportions of silt, on the contrary, recorded their lowest values in these areas. These zones correspond to the most elevated terrain where natural vegetation is prominent and accessibility is difficult for farming activities. This suggests a higher net primary production providing the input for nitrogen and carbon whose stability is reinforced by a higher clay content resulting in a higher CEC. It is widely acknowledged that SOC input is higher where substantial net primary productivity deposit occurs [83,84]. The remaining areas of lower elevation are settlement zones and cultivated areas and consequently displayed relatively medium (yellowish areas) and lower values (greenish areas) for the soil properties with some spots of high values in certain places.

### Improving predictive accuracy with remote sensing data

The results of the present study point out the potential of terrain and spectral data for soil property mapping at high resolution at local scales. However, the prediction accuracy of the different models, though satisfactory, requires further improvement. The high intrinsic spatial fluctuation in soil properties, the heterogeneity of the landscape as well as highly variable management practices by farmers are among the potential sources of noise affecting model performance. Improving prediction accuracy might require partitioning the landscape into relatively homogeneous areas based on the covariates used as predictors. The latter could be carried out by considering land surface segmentation [100] or the conditioned Latin hypercube (cLHS) [101] using the key variables driving each soil property. Moreover, though the present study considered a vast array of predictors including topographical and spectral data, a single analysis scale was applied as generally carried out in DSM. However, some studies point out the fact that the spatial distribution of soil properties is subject to various factors operating at different levels of scale [56,102]. Therefore, multi- or hyper-scale data (terrain and spectral information) which account for different spatial scales might further improve prediction as recorded in other studies such as those of Behrens et al. [103] and Miller et al. [104]. The latter authors, however, did not focus on the stratification of the land surface which in addition to multi- or hyper-scale approaches have great potential in ameliorating prediction accuracy. Further investigations are therefore required in that regards.

## Conclusion

Accurate and detailed spatial soil information is essential for environmental modelling, risk assessment and decision making. This study explored the use of high spatial resolution satellite (RapidEye and Landsat) and terrain/climatic data as well as laboratory analysed soil samples to map the spatial distribution of six soil properties–sand, silt, clay, CEC, SOC and N–in a 580 km^2^ agricultural watershed in south-western Burkina Faso. Four statistical prediction models–multiple linear regression (MLR), random forest regression (RFR), support vector machine (SVM), stochastic gradient boosting (SGB)–were tested and compared. Internal validation was conducted by cross validation while the predictions were validated against an independent set of soil samples considering the modelling area and an extrapolation area.

Results indicate that the RFR performed marginally better than the remaining models at modelling stage for most soil properties except for sand and clay for which MLR offered a better predictive ability. However, the RFR achieved a higher performance statistics for the external validations in the considered areas but not for all soil properties in the extrapolated area. Beyond the modelling area, the SVM better predicted SOC while SGB performed better for CEC and N.

The machine learning algorithms performed generally better than the MLR for the prediction of soil properties at unsampled locations. Inability of MLR to handle non-linear relationships between dependent and independent variables is believed to be the source of this limitation. Prediction accuracies from the RFR model ranged from 68% for CEC to 89% for silt.

These prediction accuracies can be deemed to be reasonable, considering the high variability in farm management practices and environmental variables in the studied watershed. Satellite data acquired during ploughing or early crop development stages (e.g. May, June) were found to be the most important spectral predictors while elevation, temperature and precipitation came up as prominent terrain/climatic variables in predicting soil properties. The shortwave and near infrared channels of Landsat8 as well as soil specific indices of redness, coloration and saturation were prominent spectral channels.

The accuracies obtained in this study are promising for future local scale digital soil mapping efforts in data poor regions such as West Africa, considering the increasing availability of free high resolution remote sensing data. The use of remote sensing data can reduce soil sampling efforts and therefore reduce soil mapping costs. Further research is, however, required on the effect of high variability in farm management practices and environmental variables on the accuracy of digital soil maps. In addition, the potential of land surface stratification and multi- or hyper-scale analysis approaches in improving prediction accuracy are worth investigating.

## Supporting Information

S1 Dataset Soil properties data in the small and big catchment. (7Z)

S2 Dataset Soil properties data and code for R statistical software. (7Z)

S1 File Shapefiles of the data points in the small and big catchment. (7Z)
